# Intimate partner violence against adolescent girls and young women and its association with miscarriages, stillbirths and induced abortions in sub-Saharan Africa: Evidence from demographic and health surveys

**DOI:** 10.1016/j.ssmph.2021.100730

**Published:** 2021-01-12

**Authors:** Bright Opoku Ahinkorah

**Affiliations:** School of Public Health, Faculty of Health, University of Technology, Sydney, Australia

**Keywords:** Intimate partner violence, Pregnancy termination, Sub-saharan africa, Global health

## Abstract

Intimate partner violence has been associated with numerous consequences for women, including pregnancy termination. This study aimed to examine the association between intimate partner violence and pregnancy termination among adolescent girls and young women in 25 sub-Saharan African countries. Data for this study was obtained from the demographic and health surveys of 25 countries in sub-Saharan Africa, published between 2010 and 2019. A total of 60,563 adolescent girls and young women were included in this study. Binary logistic regression models were used in analyzing the data and the results were presented as crude odds ratios (CORs) and adjusted odds ratios (AORs) at 95% confidence interval (CI). The prevalence of intimate partner violence and pregnancy termination among adolescent girls and young women in the 25 countries in sub-Saharan Africa were 19% and 10.1% respectively. In all these countries, the odds of pregnancy termination was higher among adolescent girls and young women who had ever experienced intimate partner violence, compared to those who had never experienced intimate partner violence [COR = 1.60, 95% CI = 1.51–1.71], and this persisted after controlling for confounders [AOR = 1.58, 95% CI = 1.48–1.68]. However, across countries, intimate partner violence had significant association with pregnancy termination among adolescent girls and young women in Angola, Chad, Congo DR and Gabon (Central Africa); Benin, Burkina Faso, Cote D'lvoire, Gambia and Mali (West Africa); Comoros, Rwanda and Uganda (East Africa); and Malawi and Zambia (Southern Africa). The findings imply that reducing pregnancy termination among adolescent girls and young women in sub-Saharan Africa depends on the elimination of intimate partner violence. Thus, policies and programmes aimed at reducing pregnancy termination among adolescent girls and young women in sub-Saharan Africa, should pay particular attention to those who have history of intimate partner violence.

## Highlights

• It is estimated that the prevalence of violence against women during pregnancy in low-and middle-income countries is between 4 and 29%.
• Intimate partner violence plays a key role in fetal death through miscarriages, stillbirths or induced abortions.
• Intimate partner violence is associated with pregnancy termination among adolescent girls and young women in sub-Saharan Africa.
• Policies and programmes on pregnancy termination among adolescent girls and young women should pay particular attention to intimate partner violence.

## Introduction

Each pregnancy puts a woman at risk of death, but the propensity of maternal mortality is higher among women whose pregnancies are terminated either through induced abortions, miscarriages or stillbirths, compared to women who have live births (Mosfequr Rahman, 2015; Mizanur Rahman, DaVanzo, & Razzaque, 2010). Induced abortion, refers to any conception that does not result in a live birth due to direct action taken with the intention to terminate the pregnancy (MacQuarrie, Mallick, & Kishor, 2016). This can be safe or unsafe (Rigterink, Saftlas, & Atrash, 2013). Unsafe induced abortion involves terminating a pregnancy by a person that lacks the necessary skills or in an environment, not in conformity with minimal medical standards, or both (Haddad & Nour, 2009). The World Health Organisation defines stillbirth as the death of the foetus in the uterus before birth, at or after 28-week gestational age (World Health Organisation, 2020). Conversely, miscarriage has been defined as fetal death that occurs spontaneously prior to the 28th week of gestation (Frederiksen et al., 2018).

Globally, out of the 121 million unintended pregnancies that occurred between 2015 and 2019, 61% ended in abortion, translating to 73 million abortions per year (Bearak et al., 2020). In 2015, 2.6 million stillbirths were recorded worldwide, with more than 7178 deaths a day (World Health Organisation, 2020).

Studies have shown that the incidence of spontaneous abortion is between 10 and 20% globally (Cohain, Buxbaum, & Mankuta, 2017; Moradinazar et al., 2020). Majority of induced abortions, stillbirths and miscarriages occur in low-and middle-income countries (LMICs) and the sub-Saharan African region (Bearak et al., 2020). Studies have also found that the risk of pregnancy termination is high among adolescent girls and young women (AGYW) compared to older women (Leppälahti, Gissler, Mentula, & Heikinheimo, 2012; Onukwugha, Magadi, Sarki, & Smith, 2020; Sánchez-Páez & Ortega, 2019). One of the major risk factors for pregnancy termination is intimate partner violence (IPV) (Durevall & Lindskog, 2015).

IPV has been associated with numerous consequences for women, including loss of pregnancy, through stillbirths and miscarriages (Durevall & Lindskog, 2015). Other women who experience IPV also terminate their pregnancies through induced abortion because of high levels of depression, post-traumatic stress disorder, psychological distress, and suicidal thoughts (Mason & Lodrick, 2013; Nguyen et al., 2019; Ogunwale & Oshiname, 2017). It is estimated that between 4 and 29% of violence against women during pregnancy occur in LMICs including sub-Saharan Africa (SSA) (Mosfequr Rahman, 2015). IPV includes physical, sexual, and emotional abuse and controlling behaviours by an intimate partner (World Health Organization, 2012). Due to the negative consequences of IPV on maternal and child health, the United Nations Sustainable Development Goals (SDGs) 5.2 and 16.1 focus on ensuring the eradication of all forms of violence against women and girls and reducing significantly all forms of violence and related death rates everywhere by 2030, respectively (United Nations, 2015).

Considering the role IPV plays in fetal death, understanding the association between IPV and pregnancy termination, particularly in SSA where pregnancy termination and IPV are very high is essential. However, in SSA, only country-specific studies have been carried out in Cameroon (Alio et al., 2011), Ethiopia (Tiruye, Chojenta, Harris, Holliday, & Loxton, 2020), Ghana (Tenkorang, 2019), Nigeria (Bola, 2016) and Tanzania (Stöckl, Filippi, Watts, & Mbwambo, 2012) on the association between IPV and pregnancy termination. None of these studies specifically focused on AGYW, despite previous studies recording high prevalence of pregnancy termination among this cohort of women (Leppälahti et al., 2012; Onukwugha et al., 2020; Sánchez-Páez & Ortega, 2019). The current study seeks to fill this gap by examining the association between IPV and pregnancy termination among AGYW in 25 sub-Saharan African countries using recent Demographic and Health Surveys (DHS) data from 2010 to 2019. Findings from this study will form the basis for government and non-governmental organisations in the 25 sub-Saharan African countries to strengthen the implementation of policies and programs aimed at eliminating IPV and reducing pregnancy termination. These efforts will help the sub-region contribute to enhancing global maternal and child health.

## Methods

### Study design

Data for this study were obtained from the DHS of 25 countries in SSA, which employed a cross-sectional study design. DHS is a nationally representative survey that gather data on a number of health indicators including intimate partner violence and pregnancy termination across LMICs. These surveys are mostly carried out every five years, although this period can be longer in some countries at certain points in time due to specific country conditions. Data for each survey is gathered from men and women and these are sampled using a two-stage sampling technique. The first stage involves the selection of clusters usually called enumeration areas (EAs) and the second stage is made up of the selection of households for the survey. Details on the sampling methodology and data collection used by the DHS are published elsewhere (Corsi, Neuman, Finlay, & Subramanian, 2012). In this study, the inclusion criteria was countries whose datasets were published between 2010 and 2019 and had information on the DHS domestic violence modules and pregnancy termination. Based on the inclusion criteria, the DHS of 26 countries were initially identified. However, only the DHS of 25 countries had data on IPV and pregnancy termination. The excluded country was Mozambique, which had no data on pregnancy termination. Again, based on eligible respondents for the domestic violence modules (Hindin, Kishor, & Ansara, 2008; MacQuarrie et al., 2016), only ever married, currently married and cohabiting AGYW were included. In all, 60,563 ever married, currently married and cohabiting AGYW who had complete information on intimate partner violence and pregnancy termination were included in this study. The countries included in this study are shown in Table 1. The manuscript was prepared in line with the Strengthening Reporting of Observational studies in Epidemiology (STROBE) reporting guidelines (Von Elm et al., 2014).

**Table 1:** Sample distribution by country.

Survey Countries	Survey Year	Weighted Sample	Percentage
**Central Africa**			
Angola	2016	2468	4.08
Cameroon	2018	2023	3.34
Chad	2015	4220	6.97
Congo DR	2013–14	3490	5.76
Gabon	2012	1094	1.81
**West Africa**			
Benin	2018	2680	4.43
Burkina Faso	2010	3826	6.32
Cote D'lvoire	2011–12	1605	2.65
Gambia	2013	1888	3.12
Mali	2018	2493	4.12
Nigeria	2018	6537	10.79
Sierra Leone	2019	1931	3.19
Togo	2013–14	1160	1.92
**East Africa**			
Burundi	2017	1976	3.26
Comoros	2012	839	1.39
Ethiopia	2016	2650	4.37
Kenya	2014	1978	3.27
Rwanda	2015	1121	1.85
Tanzania	2016	2476	4.09
Uganda	2016	3829	6.32
**Southern Africa**			
Malawi	2016	5608	9.26
Namibia	2013	508	0.84
South Africa	2016	334	0.55
Zambia	2018	2121	3.50
Zimbabwe	2015	1707	2.82

## Study variables

### Outcome variable

Pregnancy termination was the dependent variable in this study. To derive this variable, survey participants were asked “have you ever had a pregnancy terminated?” In the DHS, pregnancy termination refers to stillbirths, miscarriages or induced abortions (MacQuarrie et al., 2016). Two responses emerged from this question “No” and “Yes”. These two responses were coded as follows: Yes = 1 and No = 0. The coding of the responses was based on previous studies (Dickson, Adde, & Ahinkorah, 2018; Seidu, Ahinkorah, Agbemavi, Amu, & Bonsu, 2019; Seidu et al., 2020).

### Key explanatory variable

The main explanatory variable for the study was IPV. This variable was generated from three key variables (sexual violence, emotional violence and physical violence) based on a number of questions in the domestic violence module, where questions are based on a modified version of the conflict tactics scale (Kishor, 2005; Straus, 1979). On physical violence, each respondent was asked whether her last partner ever pushed her; shook or threw something at her; slapped her; punched her with his fist or something harmful; kicked or dragged her; strangled or burnt her; threatened her with a knife, gun or other weapons; and twisted her arm or pulled her hair. Questions on emotional violence focused on whether a respondent's last partner ever: humiliated her; threatened to harm her; and insulted or made her feel bad. On sexual violence, respondents were asked whether the partner ever physically forced them into unwanted sex; whether the partner ever forced them into other unwanted sexual acts; and whether the respondent has been physically forced to perform sexual acts she did not want to. For each of these questions, the responses were ‘yes’ and ‘no’. A respondent who had experienced at least one of the violent acts was considered as ever experienced physical, emotional or sexual violence. IPV was generated from all the questions asked on physical, emotional and sexual violence, with respondents experiencing at least one of these violent acts regarded as ever had IPV and otherwise.

### Covariates

Based on the findings of previous studies on predictors of pregnancy termination (Dickson et al., 2018; Seidu et al., 2019, 2020), six variables (age, level of education, place of residence, wealth quintile, parity, media exposure) were considered in this study as covariates. Apart from media exposure, the remaining variables were pre-coded in the DHS datasets. Media exposure was created from three variables on the frequency of watching television, listening to radio or reading newspaper/magazine. Each of these were categorised into not at all, less than once a week and at least once a week. These were re-categorised into ‘No’ (not at all) and “Yes” (less than once a week and at least once a week).

### Data analysis

Data analyses were carried out using Stata version 14.0. First, the prevalence of IPV and the proportion of AGYW who had ever experienced pregnancy termination were calculated from frequencies and percentages and presented using bar charts. Next, Pearson's chi-square test of independence was used to examine the independent associations between physical violence, emotional violence, sexual violence, and IPV and pregnancy termination in each of the 25 countries included in this study. Finally, the effect of IPV on pregnancy termination in each of the 25 countries was assessed through bivariate and multivariable binary logistic regression models. The results were presented as crude odds ratios (CORs) and adjusted odds ratios (AORs), at 95% confidence intervals (CIs). The women's sample weights for the domestic violence module (d005/1,000,000) were applied to obtain unbiased estimates, according to the DHS guidelines and the survey command (SVY) in Stata was used to adjust for the complex sampling structure of the data in the regression analyses.

## Results

### Prevalence of IPV among adolescent girls and young women in sub-Saharan Africa

**Fig. 1:**
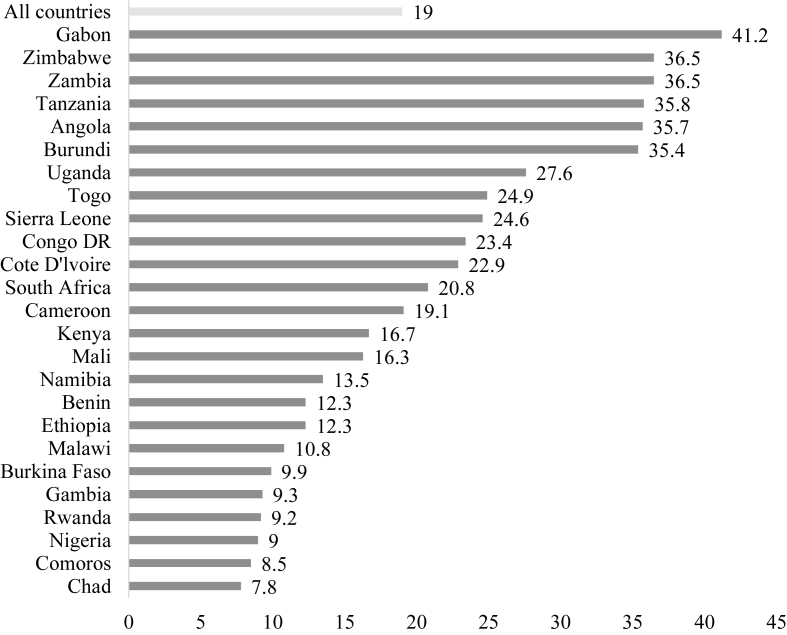
Prevalence of IPV among adolescent girls and young women in sub-Saharan Africa.

The prevalence of IPV among AGYW in the 25 countries was 19%, with the highest prevalence in Gabon (41.2%) and the lowest prevalence in Chad (7.8%) (Fig. 1).

### Prevalence of pregnancy termination among adolescent girls and young women in sub-Saharan Africa

**Fig. 2:**
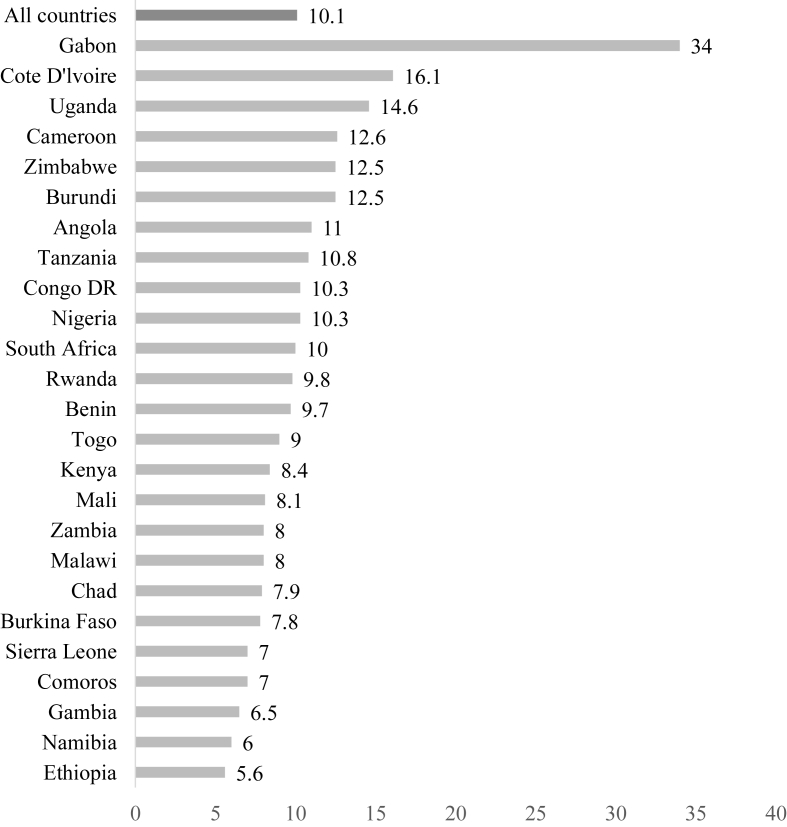
Proportion of adolescent girls and young women who have ever had a pregnancy termination in sub-Saharan Africa by country.

In the 25 countries, the prevalence of pregnancy termination among AGYW was 10.1%, ranging from as high as 34% in Gabon to as low as 5.6% in Ethiopia (Fig. 2).

### Distribution of pregnancy termination across IPV

Table 2 shows the distribution of pregnancy termination across physical violence, emotional violence, sexual violence and IPV by countries. IPV had significant association with pregnancy termination in 15 (Angola, Benin, Burkina Faso, Chad, Comoros, Congo DR, Cote D'lvoire, Gabon, Malawi, Mali, Nigeria, Sierra Leone, Togo, Uganda, and Zambia) out of the 25 countries. In these countries, pregnancy termination was higher among AGYW who had ever experienced IPV.

**Table 2:** Physical, emotional, sexual, intimate partner violence and pregnancy termination by countries.

Countries	Pregnancy termination		p-values	Pregnancy termination		p-values	Pregnancy termination		p-values	Pregnancy termination		p-values
	Never experienced PV	Ever experienced PV		Never experienced EV	Ever experienced EV		Never experienced SV	Ever experienced SV		Never experienced IPV	Ever experienced IPV	
**Central Africa**												
Angola	9.7	14.1	0.008	8.6	18.3	<0.001	10.8	14.3	0.018	9.0	14.6	0.001
Cameroon	12.2	14.9	0.056	10.7	15.1	0.220	11.0	19.6	0.002	11.9	15.2	0.053
Chad	7.4	15.7	<0.001	6.1	14.4	<0.001	6.8	16.4	0.002	7.4	13.8	<0.001
Congo DR	9.7	13.0	<0.001	9.2	12.3	0.028	7.9	17.3	<0.001	9.9	11.6	0.001
Gabon	33.6	34.7	0.011	32.3	34.1	0.030	33.9	28.1	0.078	33.6	34.5	0.003
**West Africa**												
Benin	9.3	16.9	0.007	8.2	14.5	0.006	9.6	15.7	0.070	9.1	13.9	0.003
Burkina Faso	7.4	13.1	0.004	7.5	12.5	0.008	7.8	11.8	0.199	7.2	12.4	0.001
Cote D'lvoire	15.1	19.9	0.038	16.0	18.8	0.059	15.8	27.9	<0.001	14.9	19.8	0.003
Gambia	6.3	9.3	0.167	7.0	10.2	0.070	6.9	26.6	0.001	6.1	10.7	0.056
Mali	7.5	12.5	0.011	7.6	11.6	0.018	8.4	12.5	0.207	7.4	11.6	0.007
Nigeria	10.2	12.6	0.148	9.5	11.3	0.024	9.4	16.3	0.104	10.1	12.1	0.033
Sierra Leone	6.9	7.6	0.650	6.4	9.1	0.194	6.8	17.6	0.150	6.7	8.0	0.278
Togo	7.8	16.8	0.001	8.0	13.2	0.013	9.1	12.1	0.292	7.4	14.0	<0.001
**East Africa**												
Burundi	12.4	12.6	0.382	12.0	13.5	0.152	11.8	13.8	0.063	12.3	12.8	0.373
Comoros	6.5	18.6	0.001	6.8	15.5	0.013	7.0	37.4	0.001	6.2	16.2	<0.001
Ethiopia	5.5	7.0	0.305	6.7	5.6	0.858	6.7	4.0	0.402	5.5	13.6	0.355
Kenya	8.1	10.2	0.524	5.9	12.4	0.025	7.4	9.0	0.336	7.9	10.5	0.239
Rwanda	9.3	16.5	0.028	10.7	14.4	0.336	10.6	20.7	0.141	9.3	14.8	0.059
Tanzania	10.0	13.0	0.168	10.0	12.1	0.080	9.8	16.1	0.142	9.9	12.4	0.219
Uganda	13.5	19.2	<0.001	12.1	18.3	0.001	13.3	18.0	0.069	13.5	17.5	0.001
**Southern Africa**												
Malawi	7.8	10.7	0.040	6.6	9.6	0.011	6.4	11.4	0.015	7.8	9.1	0.027
Namibia	5.7	8.7	0.447	5.6	13.7	0.116	7.5	11.6	0.911	5.3	10.7	0.200
South Africa	9.4	13.3	0.413	5.5	21.9	0.081	7.6	25.4	0.203	8.5	15.5	0.728
Zambia	7.3	9.6	0.082	7.7	9.9	0.198	7.6	11.8	0.005	6.9	9.8	0.021
Zimbabwe	12.3	13.0	0.846	10.3	15.4	0.005	11.5	14.8	0.321	11.8	13.6	0.355

Note: Pearson chi-square test was used to obtain p-values; PV=Physical violence; EV = Emotional violence; SV=Sexual violence; IPV=Intimate partner violence.

### Association between IPV and pregnancy termination among adolescent girls and young women in sub-Saharan Africa

In all the 25 countries considered in this study, the odds of pregnancy termination was higher among AGYW who had ever experienced IPV [COR = 1.60, 95% CI = 1.51–1.71], and this persisted after controlling for the covariates [AOR = 1.58, 95% CI = 1.48–1.68]. In the adjusted model, IPV had significant association with pregnancy termination in Angola [AOR = 1.60, 95% CI = 1.21–2.13], Chad [AOR = 1.85, 95% CI = 1.27–2.69], Congo DR [AOR = 1.48, 95% CI = 1.17–1.89] and Gabon [AOR = 1.45, 95% CI = 1.12–1.89] in Central Africa. In West Africa, the odds of pregnancy termination was higher among AGYW who had ever experienced IPV in Benin [AOR = 1.68, 95% CI = 1.18–2.38], Burkina Faso [AOR = 1.98, 95% CI = 1.40–2.79], Cote D'lvoire [AOR = 1.59, 95% CI = 1.02–3.46], Gambia [AOR = 1.74, 95% CI = 1.04–2.91] and Mali [AOR = 1.65, 95% CI = 1.15–2.39]. In East Africa, IPV had significant association with pregnancy termination in Comoros [AOR = 2.87, 95% CI = 1.43–5.77], Rwanda [AOR = 1.88, 95% CI = 1.02–3.46] and Uganda [AOR = 1.39, 95% CI = 1.14–1.70]. Finally, in Southern Africa, IPV had significant association with pregnancy termination in Malawi [AOR = 1.41, 95% CI = 1.05–1.89] and Zambia [AOR = 1.39, 95% CI = 1.01–1.90] (see Model II of Table 3).

**Table 3:** Multivariable models showing the association between IPV and pregnancy termination among adolescent girls and young women in sub-Saharan Africa.

Countries	Model I	Model II
	COR [95% CI]	AOR [95% CI]
Central Africa		
Angola	1.59** [1.20–2.10]	1.60** [1.21–2.13]
Cameroon	1.35 [0.99–1.82]	1.34 [0.98–1.83]
Chad	2.07*** [1.42–3.00]	1.85** [1.27–2.69]
Congo DR	1.47** [1.16–1.87]	1.48** [1.17–1.89]
Gabon	1.46** [1.14–1.88]	1.45** [1.12–1.89]
**West Africa**		
Benin	1.67** [1.18–2.36]	1.68** [1.18–2.38]
Burkina Faso	1.76** [1.26–2.46]	1.98*** [1.40–2.79]
Cote D'lvoire	1.57** [1.16–2.12]	1.59** [1.16–2.17]
Gambia	1.62 [0.98–2.67]	1.74* [1.04–2.91]
Mali	1.64** [1.14–2.35]	1.65** [1.15–2.39]
Nigeria	1.31* [1.02–1.68]	1.28 [0.99–1.65]
Sierra Leone	1.24 [0.84–1.85]	1.22 [0.82–1.84]
Togo	2.21*** [1.43–3.40]	2.52 [1.60–3.97]
**East Africa**		
Burundi	1.14 [0.85–1.52]	1.13 [0.84–1.52]
Comoros	3.25** [1.67–6.31]	2.87** [1.43–5.77]
Ethiopia	1.13 [0.69–1.85]	1.14 [0.69–1.88]
Kenya	1.27 [0.85–1.90]	1.26 [0.84–1.90]
Rwanda	1.76 [0.97–3.17]	1.88* [1.02–3.46]
Tanzania	1.18 [0.91–1.54]	1.22 [0.93–1.60]
Uganda	1.37** [1.13–1.66]	1.39** [1.14–1.70]
**Southern Africa**		
Malawi	1.38* [1.04–1.84]	1.41* [1.05–1.89]
Namibia	1.76 [0.73–4.20]	1.97 [0.80–4.86]
South Africa	1.17 [0.48–2.88]	1.10 [0.43–2.83]
Zambia	1.43* [1.05–1.94]	1.39* [1.01–1.90]
Zimbabwe	1.16 [0.85–1.59]	1.30 [0.94–1.80]
**All countries**	1.60***[1.51–1.71]	1.58***[1.48–1.68]

Model 1: unadjusted model examining the independent association of IPV and pregnancy termination; Model 2: adjusted for socio-demographic factors (age, educational level, residence, wealth quintile, parity and media exposure); AOR is the adjusted odds ratio, UOR is the unadjusted odds ratio, ref is the reference; Exponentiated coefficients; 95% confidence intervals in brackets.

*p < 0.05, **p < 0.01, ***p < 0.001.

## Discussion

Globally, the majority of unintended pregnancies that occurred between 2015 and 2019 ended in abortion and this translates to 73 million abortions per year (Bearak et al., 2020). The high prevalence of abortion has been attributed to IPV (Durevall & Lindskog, 2015). In this study, the association between IPV and pregnancy termination among AGYW was assessed. The prevalence of IPV and pregnancy termination in the 25 countries were 19% and 10.1% respectively. The highest prevalence of IPV and pregnancy termination among AGYW were observed in Gabon. In all the 25 countries considered in this study, the odds of pregnancy termination was higher among AGYW who had ever experienced IPV.

Gabon was identified as the country with the highest prevalence of IPV (41.2%) and pregnancy termination (34%) among AGYW in SSA. In terms of IPV, previous studies have found Gabon as one of the countries with the highest prevalence of IPV (Ahinkorah, Dickson, & Seidu, 2018; Coll, Ewerling, García-Moreno, Hellwig, & Barros, 2020). However, whereas Coll et al. (2020) found a prevalence of 31.2%, the study by Ahinkorah et al. (2018) found an IPV prevalence of 54.2%. The differences in prevalence of IPV in the current study and the study by Coll et al. (2020) and Ahinkorah et al. (2018) could be due to the differences in sample sizes, survey years and target population. For instance, whereas the current study focuses on AGYW, the study by Coll et al. (2020) and Ahinkorah et al. (2018) focused on all women of reproductive age. The high prevalence of IPV among AGYW in Gabon could be associated with the high rates of forced and early marriages in Gabon (UNICEF, 2018). Other reasons for the high IPV prevalence among AGYW in Gabon could be linked to the high poverty rates in the country (UNICEF, 2016), which make most of the AGYW in early and forced marriages dependent on their male partners, creating a window of opportunity for them to be abused physically, sexually and emotionally. The implication of the findings is that the elimination of IPV among AGYW in Gabon can be achieved by eliminating forced and early marriages, which can predispose AGYW to IPV. Once forced into marriages, the tendency of getting mistimed and unwanted pregnancies, leading to pregnancy termination is high and this may explain the high prevalence of pregnancy termination among AGYW in Gabon.

The pooled data showed a significant association between IPV and pregnancy termination in the 25 countries considered in this study. However, stratification of the association between the two phenomena by country showed that significant associations between IPV and pregnancy termination among AGYW existed in Benin, Burkina Faso, Cote D'lvoire, Gambia and Mali (West Africa); Comoros, Rwanda and Uganda (East Africa); Chad, Congo DR and Gabon (Central Africa); and Malawi and Zambia (Southern Africa). Similar findings have been observed in other specific countries within the sub-region such as Cameroon (Alio et al., 2011), Ethiopia (Tiruye et al., 2020), Ghana (Tenkorang, 2019), Nigeria (Bola, 2016) and Tanzania (Stöckl et al., 2012) in confirmation of the significant association between IPV and pregnancy termination in SSA. Beyond SSA, other studies have also found a positive association between IPV and pregnancy termination (Afiaz, Biswas, Shamma, & Ananna, 2020; Devries et al., 2010; Khan, Dongarwar, Aliyu, & Salihu, 2019; Rahman, 2015). This finding calls for the strengthening of the implementation of policies and programs aimed at eliminating IPV and reducing pregnancy termination and help the sub-region contribute to enhancing global maternal and child health.

### Policy and public health implications

The results of this study are crucial for policy and practice regarding the elimination of IPV in SSA in alignment with the SDG's target by 2030. The achievement of the SDG's target of eradicating IPV by the year 2030, depends on a timely intervention and policy, designed for countries in SSA. Policies and programmes by governmental and non-governmental organisations in SSA should focus on the prevention of IPV, especially in countries with high prevalence of IPV. Such policies and programmes need to be supported by a legal framework that provides educational and economic support for women and make available health information and services. Again, laws prohibiting IPV should be enacted in sub-Saharan African countries where such legal provisions are lacking. Also, there is a need for strict enforcement of laws prohibiting IPV in countries where such laws exist. Finally, community sensitisation efforts by the various governments targeting various stakeholders such as community and religious leaders on the effects of IPV are also crucial.

### Strengths and **limitations**

The strength of this study is the use of relatively large datasets that are nationally representative in analysing the association between IPV and pregnancy termination among AGYW. Again, the statistical analyses performed using the large sample in this study support the accuracy of the findings. Despite these strengths, it is worth acknowledging the limitations inherent in this study. First, the surveys used in this study were based on cross-sectional data, and hence, causal interpretations of the findings cannot be established. Second, both IPV and pregnancy termination were self -reported, and as a result, there is the possibility of under-and over-reporting of data (Ahinkorah, 2020a, 2020b, 2020c).

## Conclusion

In this study, a significant association between IPV and pregnancy termination among AGYW in SSA was identified. The findings imply that reducing pregnancy termination among AGYW in SSA depends on the elimination of IPV. Thus, policies and programmes aimed at reducing pregnancy termination among AGYW should pay particular attention to those who have history of IPV. These policies and programmes can contribute to enhancing maternal and child health in SSA.

## Financial disclosure

There is no funding for this study.

## Author statement

Conceptualization: BOA.

Methodology: BOA.

Software: BOA.

Data curation: BOA.

Formal analysis: BOA.

Writing- Original draft preparation: BOA.

Validation: BOA.

Writing- Reviewing and Editing: BOA.

## Ethical statement

Ethical permissions were not required for this study since DHS datasets which is publicly available was used. Institutions that commissioned, funded, or managed the surveys were responsible for ethical procedures. ICF international as well as an Institutional Review Board (IRB) in each respective country approved all the DHS surveys in line with the U.S. Department of Health and Human Services regulations for the protection of human subjects. Data is available on https://dhsprogram.com/data/available-datasets.cfm.

## Declarations of competing interest

None.
